# Progressive multifocal leucoencephalopathy in an immunocompetent patient with favourable outcome. A case report

**DOI:** 10.1186/1471-2377-10-32

**Published:** 2010-05-18

**Authors:** Halvor Naess, Solveig Glad, Anette Storstein, Christine H Rinaldo, Sverre J Mørk, Kjell-Morten Myhr, Hans Hirsch

**Affiliations:** 1 Department of Neurology, Haukeland University Hospital, N-5021 Bergen, Norway; 2 Department of Microbiology and Infection Control, University Hospital of North Norway, N-9038 Tromsø, Norway; 3 Department of Pathology, Haukeland University Hospital, N-5021 Bergen, Norway; 4 Div. Molecular Diagnostics, Clinical & Transplantation Virology, Institute for Medical Microbiology, Petersplatz 10, CH-4003 Basel, Switzerland

## Abstract

**Background:**

To report the clinical course of PML in an apparently immunocompetent patient treated with cidofovir.

**Case Presentation:**

A 35-year-old immunocompetent man who developed progressive hemianopsia, aphasia, and limb weakness underwent repeated MRI scans of the brain, spinal fluid analyses, and brain biopsy. Before diagnosis was established based on brain biopsy, he was consecutively treated with methylprednisolone, acyclovir, ceftriaxone and plasmapheresis, but he deteriorated rapidly suggestive of the immune reconstitution inflammatory syndrome (IRIS). He started to recover two weeks after the initiation of treatment with cidofovir and has had no relapse at 3 1/2 years of follow-up. MRI has shown marked improvement.

**Conclusions:**

PML should be considered in immunocompetent patients with a typical clinical course and MRI findings compatible with PML. Treatment with cidofovir should be considered as early as possible in the disease course.

## Background

Progressive multifocal leukoencephalopathy (PML) which is caused by the JC virus (JCV), is a rare and usually fatal demyelinating disease of the central nervous system typically occurring in severely immunosuppressed patients [1]. The diagnosis of PML is presumptive when based on clinical or radiological evidence and the detection of JCV DNA by polymerase chain reaction (PCR) in the cerebrospinal fluid (CSF). The diagnosis is definitive by detection of viral protein or DNA by immunohistochemistry or *in situ *hybridisation of brain biopsies, respectively. While the JCV genomes of urine isolates usually have an archetypal regulatory region, genomes detected in the CSF and brains from PML patients have always a rearranged viral regulatory region. Even though the majority of PML cases are found in HIV infected patients, cases have been diagnosed in patients with other cellular immunodeficiencies due to haematological malignancy, chemotherapy, organ transplantation, lymphocyte depletion as well as systemic lupus erythematosus [1]. Increasing occurrence of PML in patients exposed to monoclonal antibody therapy such as natalizumab [2], rituximab [3], and efalizumab have been reported [4].

PML is often fatal [5], but prolonged survival has been reported during antiviral treatment with cidofovir [6-10]. No definitive guidelines for treatment of PML have been established. The treatment is often complicated by the immune reconstitution inflammatory syndrome (IRIS) [11,12]. We report an immunocompetent man with PML probably complicated with IRIS who was successfully treated with cidofovir.

## Case presentation

A 35-years-old man was admitted to the Department of Neurology, Haukeland University Hospital in Bergen, Norway because of increasing problems with reading during the last four weeks. Apart from surgery for appendicitis 16 years earlier the patient was previously healthy. On admission, the neurological examination was normal except for a bilateral lower right-sided quadrant anopsia.

**Figure 1:**
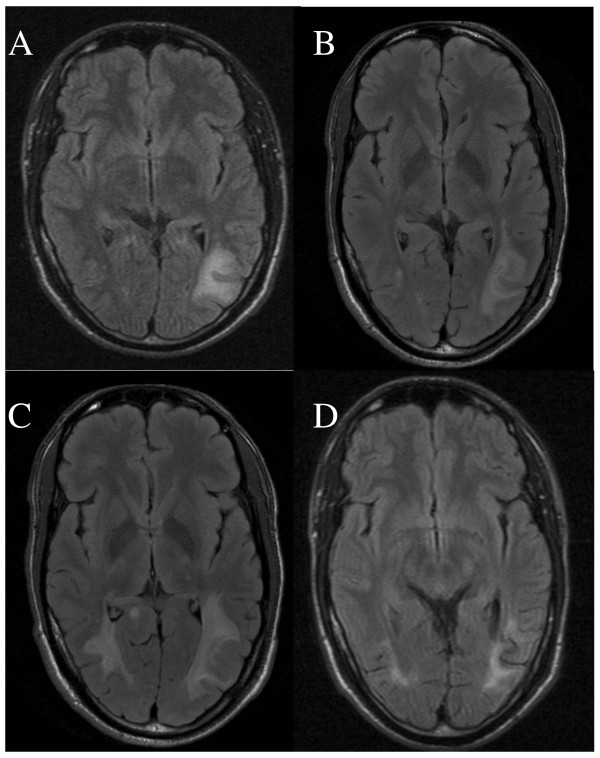
**MRI on admission and follow-up**. A. MRI (flair T2) performed on admission showing a white matter lesion in the parieto-occipital region on the left side. B. MRI (flair T2) showing progression of the white matter lesion 10 days after admission. C. MRI (flair T2) showing progression of the white matter lesions in both hemipheres 3 1/2 weeks after onset of treatment with cidofovir. D. MRI (flair T2) shower regression of the white matter lesions in both hemispheres 6 months after onset of treatment with cidofovir.

Magnetic resonance imaging (MRI) showed occipital white matter lesions mainly on the left side (Figure 1A). CSF analyses were normal (PCR on JCV was not performed). Extensive haematological and immunological blood analyses were performed including electrolytes, creatinine, liver enzymes, and CRP and they were all normal. The patient remained HIV negative on repeated tests.

**Figure 2:**
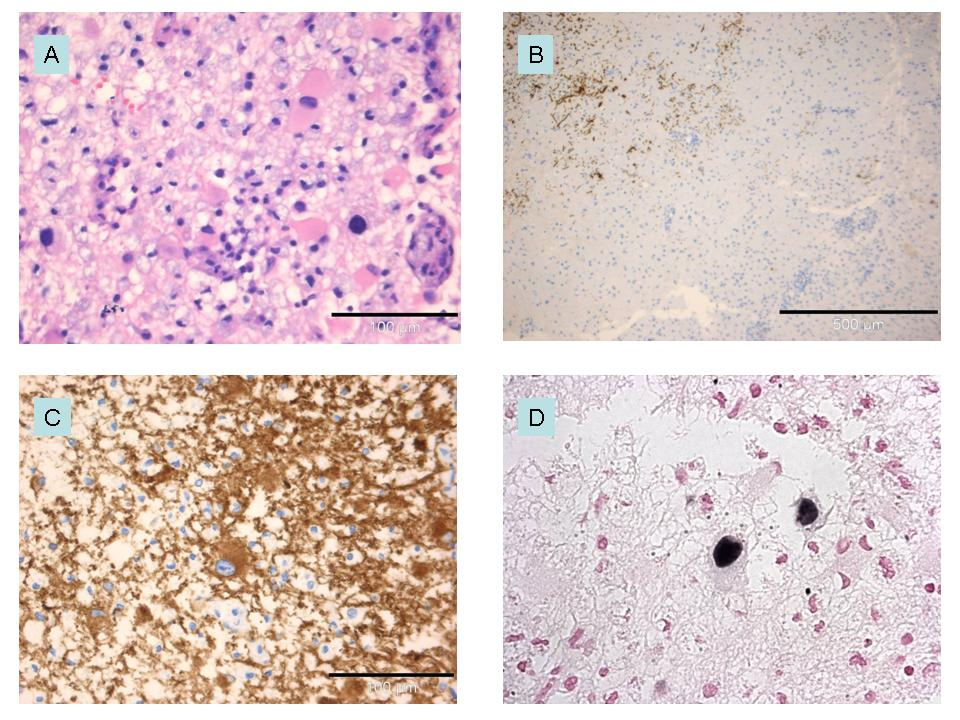
**Brain biopsy**. The biopsy specimen contained cortical grey and subcortical white matter with loose tissue texture (edema), fine caliber vacuolization, swollen, reactive astrocytes (pink cytoplasm in Panel A, hematoxylin and eosin), microglia and lipid macrophages (transformed microglia). Large pleomorphic nuclei of some astrocytes are clearly evidenced. Immunohistochemical staining for myelin basic protein (Panel B, immunoperoxidase (brown)) shows loss of myelin in the white matter lesion. Pleomorphic cells are immunopositive for astrocyte marker glial fibrillary acidic protein (Panel C, immunoperoxidase for GFAP). In-situ-hybridization of the demyelinated lesion (Panel D, original magnification ×400) shows enlarged irregular nuclei positive for JC virus.

**Figure 3:**
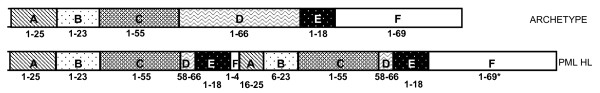
**Sequencing analysis of the JCV genome**. Schematic illustration of the archetype JCV regulatory regions/NCCR found in the urine of healthy people and the rearranged regulatory region demonstrated by the PCR in the CSF of the PML patient.

**Figure 4:**
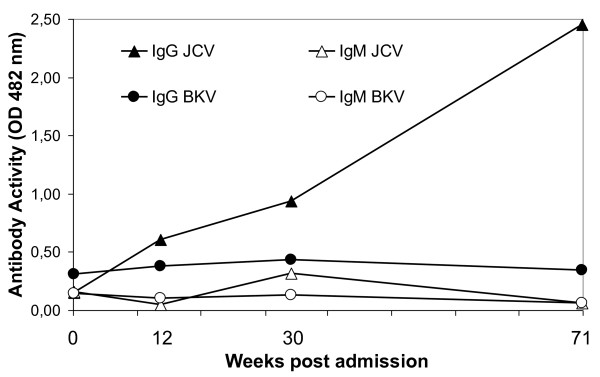
**JCV and BKV antibody titres**. JCV and BKV virus-like particle (VLP)-specific IgG levels from the time of patient administration and the following 16 months. The data were obtained retrospectively by EIA. OD 492 nm, optical density at 492 nm.

Ten days after admission the patient had developed a complete bilateral right-sided hemianopsia and slight bilateral left-sided quadrant anopsia. A new MRI showed progression of the white matter lesions (Figure 1B). The patient was consecutively treated with high dose methylprednisolone, acyclovir, ceftriaxone and plasmapheresis. However, the vision disturbances progressed and he also developed aphasia and paresis of the right arm. Four weeks after admission brain biopsy was taken from the left occipital lobe lesion. Histology showed demyelination and atypical astrocytes suggestive of PML (Figure 2A-D). PCR performed on extracted DNA from brain biopsy specimens was strongly positive for JCV. Retrospective quantitative PCR analysis of the original CSF was performed [13] and showed 2500 JCV genome copies/ml. Sequencing analysis of the JCV genome [14] showed a highly rearranged unique non-coding control region denoted PML HL (Figure 3). Retrospective enzyme immunoassay serum analysis (EIA) [14] showed JCV IgG antibodies at the time of hospitalization and the titres gradually increased at 3-months of follow-up. However, the JCV IgM levels were low and constant (Figure 4).

Treatment with intravenous cidofovir was initiated five weeks after admission. The patient received 350 mg once a week for three weeks and thereafter every fortnight for 6 months. Two weeks after onset of treatment with cidofovir the patient reported some improvement of the paresis in his right arm. Yet the MRI (Figure 1C) performed after 3 1/2 weeks treatment showed further progression compared with the MRI performed 2 weeks prior to onset of treatment with cidofovir. However, the patient continued to improve clinically, and he recovered completely from the paresis of his right arm and aphasia. After two months of treatment he also reported slight improvement of his vision.

MRI performed after 4 months of treatment showed marked regression of the white matter lesions. The patient's vision improved slightly during the next months, and the MRI (Figure 1D) showed further improvement after 6 months. The white matter lesions remained unaltered on further MRI investigations, the last one being performed 30 months after onset of treatment with cidofovir. Follow-up PCR analysis of the CSF was JCV negative seven months after onset of treatment with cidofovir.

The patient developed epilepsy 9 months after onset of symptoms.

Antinuclear antibodies, quantification of immunoglobulins (IgG 17.3 g/liter, IgA (.55 g/liter), IgM (1,3 g/liter), electrophoresis, as well as complement (C3, C4) were all normal, except for moderate reduced levels of IgA (0.55 g/L). The number of CD4 T-cells was 994 × 10^6^/liter (normal range 516 - 1494 × 10^6^/liter), the number of CD8 T-cells was 271 × 10^6^/liter (normal range 306 - 1184 × 10^6^/liter), and the number of natural killer cells (CD56) was 2.9 × 10^6^/liter (normal range 5.0 - 26.0 × 10^6^/liter). The moderately reduced numbers of CD8 T-cells and natural killer cells (CD56) were probably secondary to the treatment with methylprednisolone. It was concluded by immunologiststhat immunodeficiency was unlikely.

At the last follow-up which was 46 months after the onset of treatment with cidofovir, the patient had experienced no relapse.

## Conclusion

This case report of PML highlights several interesting points. Firstly, apparently immunocompetent persons may develop PML, and secondly, treatment with cidofovir may be successful. Thirdly, the increased progression of symptoms after initiating antiviral and antibiotic therapy, methylprednisolone and plasmapheresis, was suggestive of immune reconstitution inflammatory syndrome (IRIS). This complication may therefore follow PML treatment in apparently immunocompetent persons.

Clinical improvement within 2 weeks of the onset of cidofovir therapy suggests that cidofovir was effective. However, spontaneous recovery cannot be ruled out [15]. The progression of the MRI white matter lesions detected 3 1/2 weeks after onset of cidofovir treatment may have been related to disease progression prior to the initiation of cidofovir therapy. Others have reported clinical improvement in spite of initial worsening of lesions on MRI [16]. The MRI performed 4 months after onset of treatment showed marked improvement.

The rapid deterioration prior to treatment with cidofovir is suggestive of IRIS. IRIS is usually explained by reconstitution of a compromised immune system, followed by a strong immune response and inflammation. This may lead to a paradoxical clinical worsening of an appropriately treated infection [17]. IRIS may be seen in HIV positive PML patients who receive antiretroviral therapy [17]. or in PML patients with immunomodulatory therapy and plasmapheresis for removing the immunosupressing agent [18]. Our patient received plasmapheresis, and one might speculate whether removing the natural humoral immune response to the JCV could influence the risk of IRIS.

There are reports that cidofovir may prolong survival among immunocompromised patients with PML [6-9,19] while others have failed to confirm any effect of treatment with cidofovir [10,20]. One might speculate whether cidofovir is more effective the more immunocompetent the patient is.

In conclusion, PML should be considered in immunocompetent patients with a typical clinical course and MRI findings compatible with PML. Treatment with cidofovir should be considered as early as possible in the disease course.

## Consent

Written informed consent was obtained from the patient for publication of this case report and any accompanying images. A copy of the written consent is available for review by the Editor-in-Chief of this journal.

## Competing interests

The authors declare that they have no competing interests.

## Authors' contributions

HN investigated, treated the patient and drafted the manuscript. SG investigated the patient and contributed to important revisions of the draft. AS treated the patient and contributed to important revisions of the draft. CHR sequenced the JVC genome, and contributed to important revisions of the draft. SJM performed the histology examinations, selected histology images and contributed to important revisions of the draft. KMM contributed to conception, design and important revisions of the draft. HH performed the JCV antibody analyses. All authors read and approved the final manuscript.

## Pre-publication history

The pre-publication history for this paper can be accessed here:

http://www.biomedcentral.com/1471-2377/10/32/prepub
